# Economic Cost of Functional Neurologic Disorders: A Systematic Review

**DOI:** 10.1212/WNL.0000000000207388

**Published:** 2023-07-11

**Authors:** Brian O'Mahony, Glenn Nielsen, Sallie Baxendale, Mark J. Edwards, Mahinda Yogarajah

**Affiliations:** From the Institute of Psychiatry, Psychology & Neuroscience (B.O.M.), King's College London; Molecular and Clinical Sciences Research Institute (G.N., M.J.E.), St. George's University of London; Department of Clinical and Experimental Epilepsy (S.B., M.Y.), University College London, Institute of Neurology; Department of Neurology (S.B., M.Y.), National Hospital for Neurology and Neurosurgery; Epilepsy Society (S.B., M.Y.), Chalfont Centre for Epilepsy; and Neurology Department (M.J.E.), Atkinson Morley Regional Neuroscience Centre, St. George's University Hospitals, London, United Kingdom.

## Abstract

**Background and Objectives:**

Functional neurologic disorder (FND) represents genuine involuntary neurologic symptoms and signs including seizures, weakness, and sensory disturbance, which have characteristic clinical features, and represent a problem of voluntary control and perception despite normal basic structure of the nervous system. The historical view of FND as a diagnosis of exclusion can lead to unnecessary health care resource utilization and high direct and indirect economic costs. A systematic review was performed using Preferred Reporting Items for Systematic Reviews and Meta-Analyses guidelines to assess these economic costs and to assess for any cost-effective treatments.

**Methods:**

We searched electronic databases (PubMed, PsycInfo, MEDLINE, EMBASE, and the National Health Service Economic Evaluations Database of the University of York) for original, primary research publications between inception of the databases and April 8, 2022. A hand search of conference abstracts was also conducted. Key search terms included “functional neurologic disorder,” “conversion disorder,” and “functional seizures.” Reviews, case reports, case series, and qualitative studies were excluded. We performed a descriptive and qualitative thematic analysis of the resulting studies.

**Results:**

The search resulted in a total of 3,244 studies. Sixteen studies were included after screening and exclusion of duplicates. These included the following: cost-of-illness (COI) studies that were conducted alongside cohort studies without intervention and those that included a comparator group, for example, another neurologic disorder (n = 4); COI studies that were conducted alongside cohort studies without intervention and those that did not include a comparator group (n = 4); economic evaluations of interventions that were either pre-post cohort studies (n = 6) or randomized controlled trials (n = 2). Of these, 5 studies assessed active interventions, and 3 studies assessed costs before and after a definitive diagnosis of FND. Studies showed an excess annual cost associated with FND (range $4,964–$86,722 2021 US dollars), which consisted of both direct and large indirect costs. Studies showed promise that interventions, including provision of a definitive diagnosis, could reduce this cost (range 9%–90.7%). No cost-effective treatments were identified. Study comparison was limited by study design and location heterogeneity.

**Discussion:**

FND is associated with a significant use of health care resources, resulting in economic costs to both the patient and the taxpayer and intangible losses. Interventions, including accurate diagnosis, seem to offer an avenue toward reducing these costs.

Functional neurologic disorders (FNDs) represent genuine involuntary neurologic symptoms and signs that have characteristic clinical features and represent a problem of voluntary control and perception despite normal basic structure of the nervous system.^1^ Manifestations of FND are varied and include the following in isolation or combination: abnormal movements; weakness or paralysis; sensory loss or abnormal sensory symptoms; swallowing or speech symptoms; and epileptic-like episodes (i.e., functional seizures [FSs]).^1^ FNDs carry a significant impact on the patient's quality of life (QoL),^2,3^ and patients often present with comorbid psychiatric conditions, with both depression and anxiety occurring in up to 40% of patients with FND.^4,5^

FND has a prevalence of up to 50/100,000 and an incidence of up to 12/100,000 per year. Psychogenic nonepileptic seizure (PNES) contributes a further 1.5–4.9 per 100,000 population per year, with a prevalence of 2–33 per 100,000 population.^6^ Patients with FND make up 9% of neurology admissions,^7,8^ 16% of neurology clinic referrals,^9^ and 10%–25% of patients referred to epilepsy specialist centres.^10^ Patients with FND often require multiple consultations over several years before receiving a diagnosis of FND^11^ and then frequently re-present to emergency departments after receiving such a diagnosis.^12^ Delayed diagnosis leads to worse outcomes for patients^4^ and preventable costs, such as missed work, general practitioner and specialist appointments, and investigations. Diagnostic uncertainty amid ongoing symptoms can also lead to intangible costs, such as decreased QoL.

These costs carry a burden to patients, clinicians, and health care systems and to the economy. Indeed, patients with FND have been found to be more likely to not be working for health reasons and more likely to be receiving disability-related state financial benefits than people with other neurologic disorders.^13^

Various treatments such as physiotherapy^14^ or cognitive behavioral therapy (CBT)^15^ can lead to improvement of these symptoms and QoL. Of importance, an intervention of simply providing the patient with an accurate diagnosis, and thus explanation of their symptoms, can also improve mood and QoL^16^ and decrease health care resource utilization.^17^

The costs of FND (and other medical conditions) can be believed of as direct and indirect costs. Direct costs represent resources used for health care (e.g., cost of investigations or the time spent on assessment by a doctor), while indirect costs represent productivity losses arising from morbidity-related sickness absence (e.g., loss of employment, benefits, or the cost of childcare while hospitalized). Direct and indirect costs together constitute the economic burden of FND, which can be quantified through cost-of-illness (COI) studies. A COI study can use a top-down or a bottom-up approach. Bottom-up methods estimate costs based on data from records (or observed usage) at the service provider level, whereas top-down approaches use administrative registers of costs.^18^

Other studies of health care utilization focus on economic evaluation (EE). There are different types of EEs: cost-minimization analyses address the question of whether an intervention would result in lower health care costs. Cost-effectiveness analyses combine costs and clinical parameters, such as gained life years or recovered cases, to assess whether the intervention is cost-effective.^19^ Cost-utility analyses use quality-adjusted life years (QALYs) as their measure of effectiveness. QALYs attempt to quantify the impact of the patient's condition on the quality and quantity of life lived. Typically, cost-effectiveness analyses use the incremental cost-effectiveness ratio, which is a measure of the additional cost per unit of health gained. Whereas COI presents information only on the economic burden of a disease, EE can assist decision makers to decide toward which interventions to prioritize resources.

Given the reportedly high burden FND places on patients and society, we aimed to systematically review the health economic literature on FND. Our objectives were as follows:

1. to investigate the direct and indirect costs of FNDs and
2. to investigate whether any interventions to treat FNDs are cost-effective.

## Methods

### Criteria for Considering Studies for the Review

This study followed the methodology and guidelines set out by the Preferred Reporting Items for Systematic Reviews and Meta-Analyses checklist for systematic reviews^20^ (eAppendix 1, links.lww.com/WNL/C833). Studies were included if they reported original cost or cost-effectiveness data for FN Ds. The references of any studies whose text was read in full were screened to identify further studies. Reviews, qualitative studies, studies reporting results of other studies, qualitative studies, and any studies that were not available in English were excluded. Case reports and series were also excluded. Article were screened for inclusion by B.O.M. and M.Y., and all data were extracted by B.O.M. When a single study was published in several articles, the article reporting the largest group was used. No restrictions on age, sex, or treatment level were applied.

### Outcome Measures

The primary outcome measures were the monetary and nonmonetary costs of FND to patients and the economy.

### Search Methods for Identification of Studies

Searches were made in April 2022 from inception of the databases to April 8, 2022, in the following electronic databases: PubMed, EMBASE, MEDLINE, PsycINFO, and the National Health Service Economic Evaluations Database of the University of York and in the reference lists of identified studies. These databases contain a comprehensive list of medical literature and reports.

The following search string was used (in titles and abstracts): (“conversion disorder” OR “conversion reaction” OR psychogen* OR nonepileptic OR nonepileptic OR hysteri* OR “functional neurologic” OR “functional movement” OR “functional motor” OR “functional tremor” OR “functional sensory” OR nonorgan* OR nonorgan* OR Astasia-Abasia OR “Astasia Abasia”) AND (QALY OR “quality adjusted life year$” OR “disability adjusted life year$” OR DALY OR cost OR expense OR expenditure OR out-of-pocket OR economic OR budget OR monetary OR resource* OR consumption OR informal care).

The subject heading of conversion disorder was exploded on the Ovid platforms (Psycinfo, MEDLINE, and EMBASE). The following conference proceedings during the past 5 years were hand searched: Society of Biological Psychiatry, American Psychiatric Association, The British Neuropsychiatry Association, Royal College of Psychiatrists, Association of British Neurology, and American Academy of Neurology. Abstracts that were identified as meeting the inclusion criteria for the review had their full texts sought for assessment. B.O.M. contacted the lead author of any papers found through this method.

### Data Collection and Analysis

A record of included and excluded studies (and reasons for exclusion) was kept. Data were extracted using the DistillerSR software^21^ by B.O.M. and included study characteristics, demographics, and as economic costs such as direct health care and nonhealthcare costs, indirect costs, and QALY measurements.

A meta-analysis was not deemed appropriate, given the significant heterogeneity in the studies' cohorts, location (differing health care systems), costs included, and cost-data sources. To compare results for the noncomparator studies, costs per patient were transformed using purchasing power parities (PPPs) for gross domestic product (GDP) to US dollars (USD).^22^ The cost data of studies using year of price level before 2021 were inflated by 1% annually to calculate a common end value for the year 2021. If mean values and/or standard deviations were not reported, freely available software was used (Window Ruler) to calculate these measures from the provided graphs.

### Assessment of Paper Quality

Assessment of the overall methodological quality of EEs was informed by application of the Scottish Intercollegiate Guidelines Network Methodology checklist^23^ (eAppendix 2, links.lww.com/WNL/C834) and a checklist of methods (eAppendix 3, links.lww.com/WNL/C835). Distiller SR was used to produce quality figures based on our assessment as low, acceptable, or high quality.

### Standard Protocol Approvals, Registrations, and Patient Consents

The study protocol was registered on PROSPERO on April 8, 2022, registration number CRD42022322142. Ethics was not sought because any data collected was obtained from publicly accessible documents.

### Data Availability

Individual researchers may request collected data from the corresponding author.

## Results

Search results are shown in the Figure. Fifty-eight studies were reviewed in full text, of which 16 studies were included. Four conference abstracts were identified, and their data requested from their respective authors, of which 1 responded. Forty-two studies were excluded for reasons detailed in the Figure.

**Figure:**
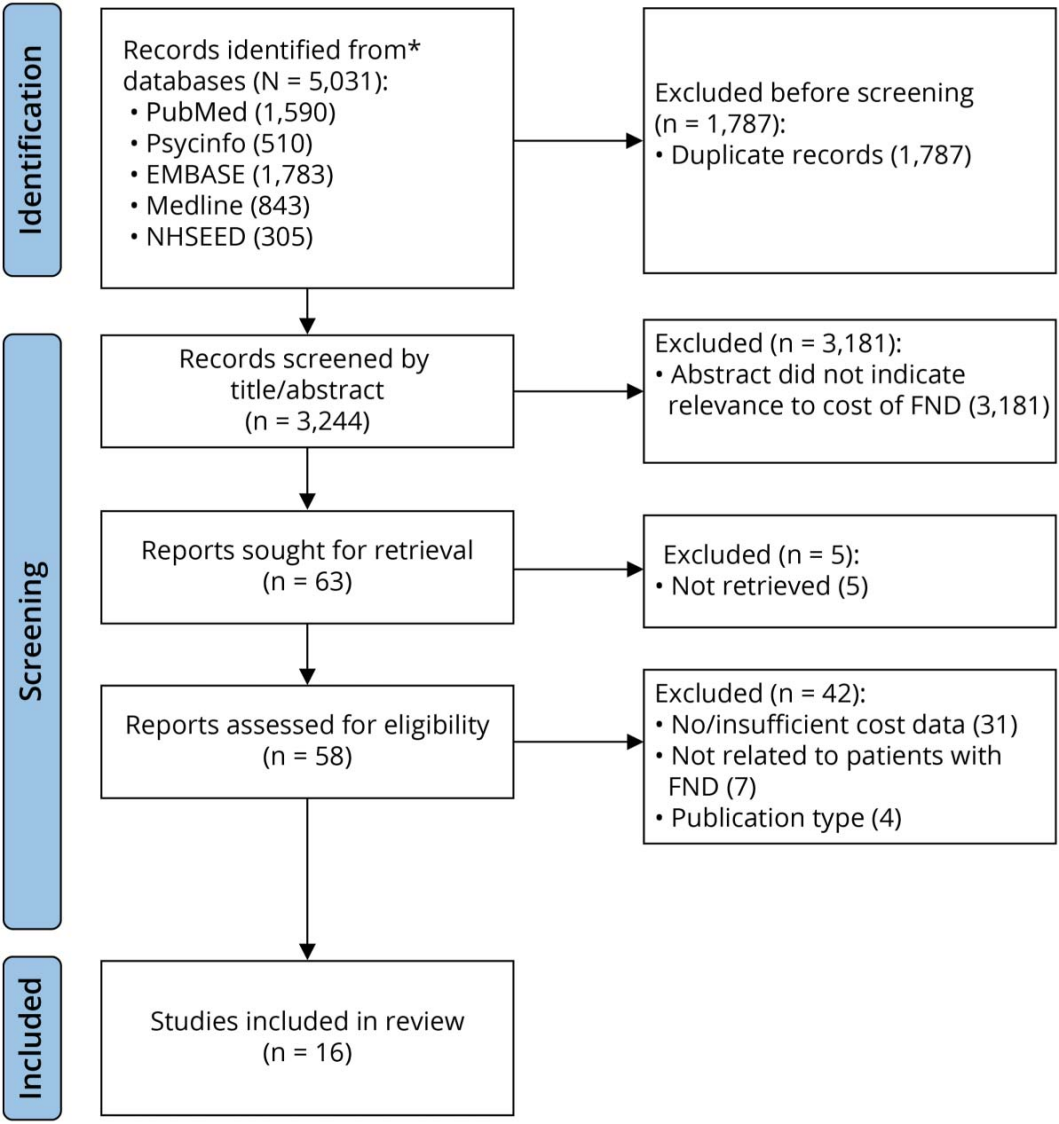
PRISMA Flowchart of Study Identification FND = functional neurologic disorder; NHSEED = National Health Service Economic Evaluation Database; PRISMA = Preferred Reporting Items for Systematic Reviews and Meta-Analyses.

### Study Quality

Of the included studies, 4 (Stephen et al.,^24^ Goldstein et al.,^25^ Jennum et al.,^26^ and Luthy et al.^27^) were deemed to be of high quality, 10 were deemed to be of acceptable quality (Deleuran et al.,^28^ Nelson-Sice et al.,^29^ Tinazzi et al.,^30^ Seneviratne et al.,^31^ Martin et al.,^32^ Ahmedani et al.,^33^ Russell et al.,^34^ Magee et al.,^35^ Nielsen et al.,^36^ Reuber et al.^37^), and 2 were deemed to be of low quality (Chemmanam et al.,^38^ Goyal et al.^39^).

### Study Characteristics

General study characteristics are summarized in Tables 1–3. The earliest study was published in 1998, the most recent in 2021. Of the included studies on COI of FND, 81% (n = 13) were published in the year 2013 or later, which perhaps indicates that the COI of FND is a topic of recent and increasing interest. Sample sizes varied from 11 to 64,138. Five studies were conducted in the United States, 4 in Great Britain, 2 in Denmark, and 1 in each of Italy, Ireland, Australia, Canada, and India. Ten studies focused on FS, 4 studies focused on FND/conversion disorder, and 2 studies focused on functional movement disorder (FMD).

**Table 1:**
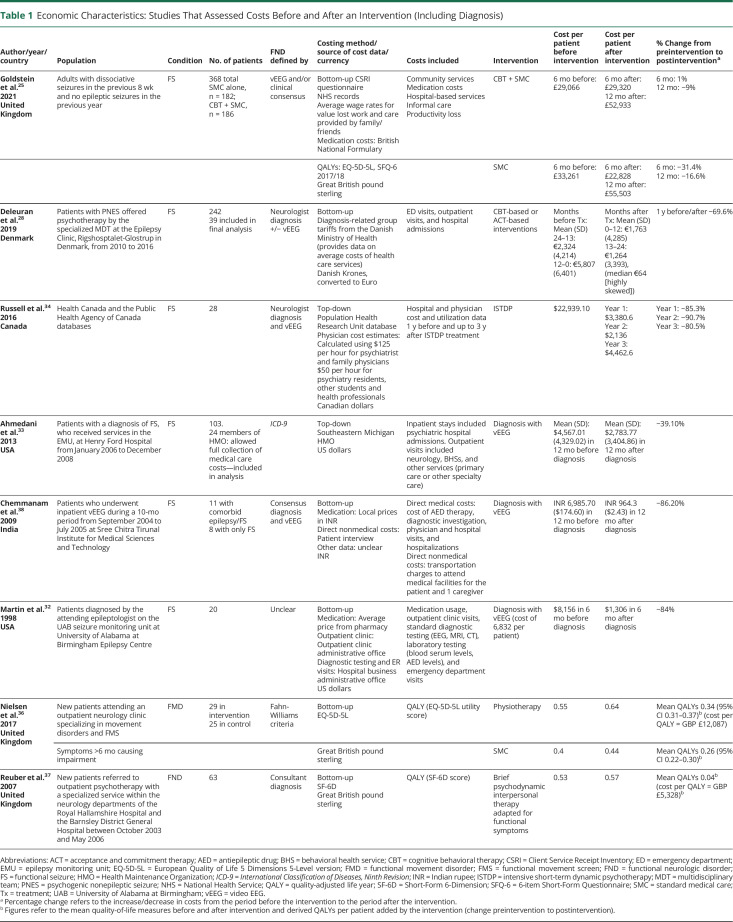
Economic Characteristics: Studies That Assessed Costs Before and After an Intervention (Including Diagnosis)

**Table 2:**
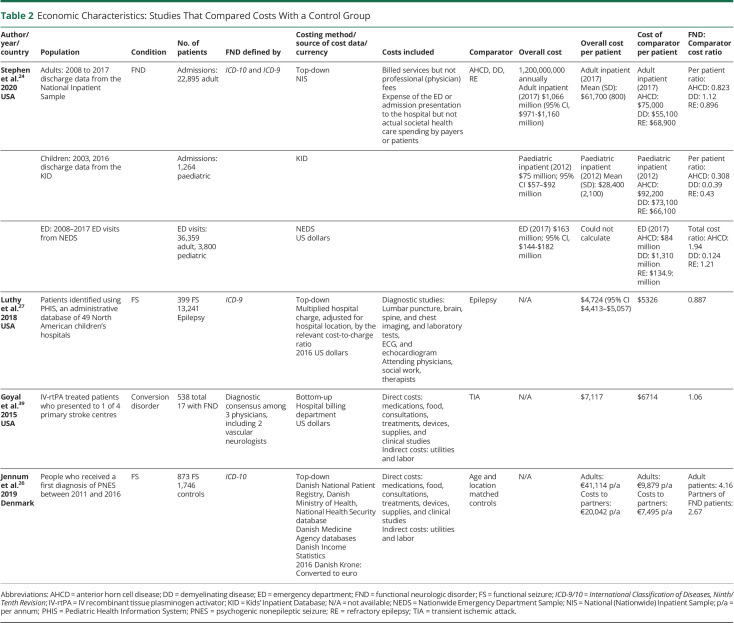
Economic Characteristics: Studies That Compared Costs With a Control Group

**Table 3:**
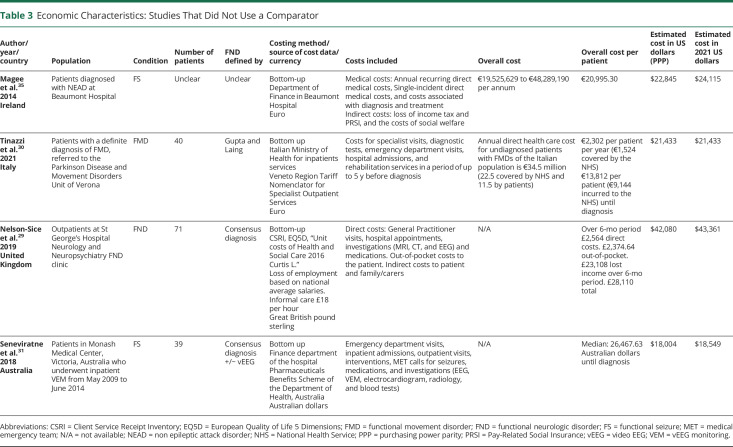
Economic Characteristics: Studies That Did Not Use a Comparator

Studies were also heterogenous for diagnostic criteria. Of the 6 studies of FND/FMD, 1 used *International Classification of Diseases, Ninth/Tenth Revision* (*ICD-9/10*) (Stephen et al.^24^), 1 used Gupta and Lang Criteria (Tinazzi et al.^30^), 1 used Fahn Williams criteria (Nielsen et al.^36^), and 3 used consensus diagnosis (Nelson-Sice et al.,^29^ Goyal et al.,^39^ Reuber et al.^37^). Of the studies of FS, 6 used the gold standard of video EEG (vEEG) (Goldstein et al.,^25^ Deleuran et al.,^28^ Russell et al.,^34^ Chemmanam et al.,^38^ Seneviratne et al.,^31^ Martin et al.^32^), 2 used *ICD-9/10* (Jennum et al.,^26^ Luthy^27^), 1 used both *ICD-10* and vEEG (Ahmedani et al.^33^), and diagnostic criteria of 1 study were unclear (Magee et al.^35^).

Study designs were made up of 3 types:

1. COI studies that were conducted alongside cohort studies without intervention, which included a comparator group, that is, another neurologic disorder (n = 4).^24,26,27,39^
2. COI studies that were conducted alongside cohort studies without intervention, which did not include a comparator group (n = 4).^29-31,35^
3. EEs of interventions that were either pre-post cohort studies (n = 6)^28,32-34,37,38^ or randomized controlled trials (RCTs, n = 2).^25,36^ Of these, 5 studies assessed active interventions, and 3 studies assessed costs before and after a definitive diagnosis of FND.

eAppendix 4 (links.lww.com/WNL/C836) displays the cost categories considered. Studies varied regarding the detail of their breakdown of costs. Eight studies assessed only hospital costs (inpatient and specialist outpatient services), with 7 of these studies focused only on hospital in-patient costs. Only 4 studies assessed medication costs outside of hospital. Three studies assessed productivity losses to the patient and informal carers resulting from their FND, while Jennum et al.^26^ assessed productivity loss regarding cost to the state. Studies also varied regarding their reporting of cost data. Although authors reported including different types of costs in their analysis, some did not give exact figures for these individual costs. eAppendix 5 (links.lww.com/WNL/C837) details what costs were explicitly reported, by paper. Two articles gave only the total overall cost per patient.

### Population Demographics

Thirteen studies investigated the costs of adults only, and 2 studies (Stephen et al.^24^ and Jennum et al.^26^) included both adults and minors with FND. Luthy et al.^27^ investigated the costs at a pediatric hospital. Adult patients' mean/median age in studies ranged between 35^25,31^ and 45.48 years,^24^ and every study that noted sex ratio reported mostly female patients, ranging from 57%^34^ to 86%.^28^

### Economic Costs

Findings are summarized in Tables 1–3. Several summary results can be derived from the economic data presented in the selected studies.

First, 8 studies assessed costs before/after an intervention, where intervention was defined as psychological-based treatments or making and communicating a robust diagnosis. Each of these 8 studies showed cost reduction, or improved QALYs, in the period after the intervention. The only study^25^ which conducted a full cost-benefit analysis was calculated the incremental cost of CBT and standard medical care (SMC) as £120,658 per QALY compared with SMC alone. This fell above the threshold for cost-effectiveness required by the National Institute for Health and Care Excellence (NICE) of under £20,000–£30,000 per QALY.^40^ Nielsen et al.'s^36^ pilot RCT of a physiotherapy intervention for patients with FMD reported a mean incremental cost per QALY gained of £12,087,^36^ while Reuber et al.'s^37^ uncontrolled pilot study reported a mean incremental cost per QALY gained of £5,328 (if QoL improvements lasted 1 year) for a brief psychodynamic intervention in patients with mixed functional neurologic symptoms.^37^

Second, in those studies that compared FND costs with those for other chronic neurologic diseases, costs were similar. Both Luthy et al.^27^ and Stephen et al.^24^ showed a lesser cost burden of PNES compared with epilepsy, although the latter study showed greater cost in emergency settings, despite the fact that Stephen et al.^24^ included only refractory epilepsy as a comparator. The only study that compared the economic costs of patients with FND with those of healthy controls (Jennum et al.^26^) showed a marked increase in costs to both patients with FND and their carers.

Third, in those studies that gave estimates of total costs to the taxpayer, Stephen et al.,^24^ Tinazzi et al.,^30^ and Magee et al.^35^ gave estimates of the total COI to their countries of $1,200,000,000 USD (hospital charge costs for all FND subtypes and all ages), €34,500,000 (direct health costs for functional motor symptoms in people older than 16 years), and €19,525,629 and €48,289,190 (direct and indirect costs for FSs in adults per annum, respectively).

Finally, overall costs varied significantly because of the studies' methodological and geographical heterogeneity; after costs were adjusted to PPPs for GDP, mean annual costs per patient of PNES ranged from $4,964 2021 USD (Luthy et al.)^27^ to $83,884 2021 USD (Goldstein et al.),^25^ while those of FN Ds ranged from $21,433 2021 USD (Tinazzi et al.)^30^ to $86,722 2021 USD (Nelson-Sice et al.).^29^

## Discussion

This systematic review of health economic studies for FND indicates the significant cost of the disorder, and the possibility to mitigate this cost. Our findings indicate 2 trends: first, that FND causes costs per patient, comparable with, or in excess of, other chronic neurologic disorders with similar symptoms (e.g., FS vs epilepsy). Second, that interventions (including making and delivering a robust diagnosis) have the potential to improve patients' health status (measured in both QALYs and symptom relief) and reliance on health care resources, with a subsequent reduction of costs. However, the heterogeneity of studies provides challenges in interpreting and comparing results.

There was significant variation in reported costs, possibly resulting from heterogeneity in diagnostic practices, differences in types of costs included, cost data sources, and study location. After costs were adjusted to PPPs for GDP, mean annual costs ranged from $4,964 2021 2021 USD (Luthy et al.)^27^ to $86,722 2021 USD (Nelson-Sice et al.).^29^ This heterogeneity of costs is also reflected in systematic reviews of the economic costs of Medically Unexplained Symptoms (mean annual costs ranging from $1,584 to $6,424 2006 USD from 1986 to 2004),^41^ multiple sclerosis (mean annual costs ranging from $13,721 to $82,080 2012 USD from 1995 to 2012),^42^ Epilepsy (mean annual direct costs ranging from £611 to €4,292 from 1992 to 2013),^43^ and Treatment-Resistant Depression (mean annual costs ranging from $3,800 to $49,000 2006 USD from 2004 to 2014).^44^

This heterogeneity limits not only comparisons of studies included in this review but also the comparison of the economic cost of FND with the economic costs of other chronic, neurologic, and psychiatric disorders. However, 2 high-quality studies included in this review (Stephen et al.^24^ and Luthy et al.^27^) reported FND and FS, respectively, as having a similar mean direct cost per patient as epilepsy. Stephen et al.^24^ also reported a similar mean direct cost per adult patient admitted with FND as with demyelinating disorders. Given that patients with FND have levels of physical disability equivalent to people with multiple sclerosis or epilepsy and higher frequencies of psychological comorbidities than those 2 disorders,^13^ one might expect similar or greater indirect and intangible costs. This provides powerful insight into the economic impact of a disorder, which has relatively limited awareness in the medical community.^45,46^

Given the high prevalence of comorbidities which occur in patients with FND,^4,5^ it is possible that these comorbidities might have contributed to the costs calculated by the articles included in this review. This lack of adjustment would have led to inflated costs being calculated for the FND cohort.^47^ Luthy et al. attempted to isolate the pure economic cost of FND through the use of an extensive exclusion criterion (of both medical and psychiatric comorbidities). The authors acknowledged that study of such a cohort likely lessened the external validity of their findings, given that the successful treatment of many chronic neurologic disorders, and especially FND, requires a holistic approach.

In those studies that assessed economic effectiveness of interventions using QALYs, there was significant variability. Part of this is due to differences in the patient population and interventions. However, in 2 studies, the patient population and intervention were similar, namely patients with FS undergoing psychological-based treatments. Despite this, there were significant differences in QALY costs. Goldstein et al.^25^ reported an incremental cost of CBT and SMC as £120,658 per QALY compared with SMC alone, while Reuber et al.^37^ reported the mean incremental cost per QALY gained as £5,328. A number of factors are likely to contribute to these widely differing figures. Reuber et al.^37^ (n = 63) reported a unit cost of treatment as £213.15, while Goldstein et al.^25^ (n = 293) reported a unit cost of £1,064. Furthermore, Reuber et al.^37^ based their analysis on clinical outcomes at 6 months, which they assumed would be the same at 12 months. If Goldstein et al.^25^ were to use clinical outcomes at 6 instead of 12 months, the cost per QALY gained would be lower because there was a greater QoL difference at that time point and a significant difference in the primary outcome measure of seizures. Finally, as Reuber et al.^37^ acknowledge, the lack of a control group in their study means that the cost-effectiveness of intervention cannot be regarded as proven in view of confounders such as placebo or regression to the mean effects. Moreover, the control arm in the study conducted by Goldstein et al.^25^ was not treatment as usual, but enhanced “standardized medical care,” a package of care greater than what is typically provided for patients with FS, involving education and counseling from neurologists and psychiatrists. This in turn would have led to a smaller difference in QALY effects in the group and therefore an underestimation of the cost-effectiveness of the intervention.

In studies without comparators, total costs varied from $18,549 to $43,661 2021 USD. Any conclusions reached from these studies is limited by their lack of a comparison group, and it is thus difficult to contextualize their reported findings.

A comprehensive COI study should include all direct and indirect health care costs and intangible costs. Most of the studies in this review included only hospital-related costs. Such studies would underestimate the true economic cost of FND. A direct comparison of inpatient admissions costs was also limited by the difference in specific costs included in the studies, for example, diagnostic imaging, medication, or multidisciplinary team consultations.

Another complication of comparing costs from studies is their setting in different countries and therefore different health care systems. Different countries have varying degrees of public health care systems, with patients carrying extra costs in more private systems. Such differences alter resource allocation by clinicians, and differences in health care systems have been shown to alter patients' use of health care resources.^48^

Countries with more extensive social supports might also affect indirect costs. Jennum et al.^26^ identified that, compared with controls, a greater proportion of people with FS and their partners received social service benefits, such as sick pay or disability pension and housing benefits. The authors reported that because of these public services, early retirement may be more common. Studies that assessed productivity loss^20,25,29,35^ reported that these costs dwarfed those of direct costs. Productivity loss is likely to vary across countries and thus affect differently on the overall economic cost of FND.

The studies in this review demonstrated the high cost of undiagnosed FND and the reduction of this cost with diagnosis. This highlights the importance of establishing an early diagnosis of FND. The possible reasons for this are 2-fold; minimization of excessive investigations and inappropriate medications,^9^ lessening the direct and indirect economic costs associated with both, while also minimizing harm to the patient; improvement of their prognosis after careful communication of a clear and robust diagnosis.^e1^ However, none of the studies reviewed have been able to discriminate between these 2 possibilities. The studies identified in this study suggest that this particularly applied to those patients with FS who receive a gold standard diagnosis by way of vEEG. In those studies that assessed treatment interventions, costs were significantly reduced after the treatment intervention, but the evidence for the cost-effectiveness of those interventions is currently more limited.

Future research in this area should ideally include a comprehensive list of direct and indirect costs to ascertain the full extent of the economic burden of FND. More studies from middle-income and low-income countries along with the inclusion of appropriate comparison groups would enable a comprehensive understanding of the global economic burden of FND.

To date, there has been no large study showing cost-effectiveness of a treatment for FND, defined by NICE as a cost per QALY below $35,000–$45,000 2009 USD across countries.^e2^ To our knowledge, only Goldstein et al. have thus far performed a comprehensive cost-effectiveness study, though costs were above NICE thresholds. Thus, rigorous cost-effectiveness studies should also be undertaken to investigate cost-effective treatments for FND. Similarly, studies should seek to distinguish the relative contributions to reduced costs after a diagnosis of FND, of the robustness of diagnostic communication, reduced inappropriate medical interventions, or improved prognosis.

As with other health economic systematic reviews, our review is faced with the limitation that studies that use top-down cost calculations would underestimate privately paid health care goods, while those using hospital charge data would, on average, overestimate the true economic cost of the disorder 2-fold.^e3^

This review highlighted a relative paucity of research into this topic. Four studies assessed indirect costs to the patient, and only 3 studies^25,36,37^ included intangible costs. Productivity loss and intangible costs, such as cost associated with stigma, have been shown to make up a significant portion of the cost of epilepsy,^e4^ and their exclusion from most of the studies in this review limits any estimate of the true burden of FND. The tertiary location of several studies meant that their population represented a severe subset of patients with FND and thus may limit the external validity of their findings. FND is a heterogeneous disorder, even in patients with the same symptoms. Treatment approaches based primarily on the presenting symptom without consideration of other comorbid problems may therefore dilute or even obscure treatment benefit for a subset of patients. This may in turn increase the associated costs per QALY of the intervention. Finally, most of the comparator studies in this review used control groups with chronic neurologic diseases (e.g., motor neuron disease, multiple sclerosis). Only a minority of such studies matched FND symptoms across groups, which should be an aim of future studies to understand relative costs more robustly (e.g., comparison of costs of FMDs with Parkinson disease).

FN Ds are associated with the significant use of health care resources, resulting in economic costs to patient and the taxpayer and intangible losses. Given that FN D is a medical condition similar to any other, we do not suggest that there should be zero cost associated with it. Rather, in this review, we have tried to explore how these costs can be moderated effectively with timely diagnosis and treatment. Interventions, including simply making a robust diagnosis, seem to offer an avenue toward reducing these costs. Significant heterogeneity exists between studies in this area, and we found a relative lack of research on indirect and intangible costs. Such costs seem to be high in FN Ds and offer a focus for further research, as do longer-term studies.

## Glossary

CBT: cognitive behavioral therapy
COI: cost of illness
FMD: functional movement disorder
FND: functional neurologic disorder
FS: functional seizure
GDP: gross domestic product
*ICD-9/10*: *International Classification of Diseases, Ninth/Tenth Revision*
NICE: National Institute for Health and Care Excellence
PNES: psychogenic nonepileptic seizure
PPP: purchasing power parity
QALY: quality-adjusted life year
QoL: quality of life
RCT: randomized controlled trial
SMC: standard medical care
USD: US dollar
vEEG: video EEG
